# Creating large ^60^Co-γ populations for functional genomics and breeding in wheat

**DOI:** 10.3389/fpls.2025.1760299

**Published:** 2026-01-26

**Authors:** Qunqun Hao, Simeng Ma, Jifa Zhang, Yuhai Wang, Wenqiang Wang

**Affiliations:** 1 College of Life Sciences, Zaozhuang University, Zaozhuang, China; 2 Jinan Key Laboratory of Biological Breeding, Spring Valley Agriscience Co. Ltd., Jinan, China; 3 State Key Laboratory of Wheat Improvement, College of Agronomy, Shandong Agricultural University, Tai’an, China

**Keywords:** ^60^Co-γ, breeding, genomics, germplasm, wheat

## Abstract

Wheat (*Triticum aestivum* L.) serves as a critically important staple crop worldwide, and mutation breeding through Cobalt-60 (^60^Co-γ) radiation has been widely adopted as an effective strategy for genetic improvement. In this study, ten wheat cultivars from Shandong, Henan, and Hebei were subjected to ^60^Co-γ irradiation to develop an M_2_ mutant population comprising 10,350,000 lines. Systematic screening M_2_ mutant population under natural conditions identified 158 freezing-tolerant mutants, 441 saline-alkali-tolerant mutants, and >5,000 mutants with changed yield or quality traits. This population represents a valuable genetic resource for collaborative research and provides a powerful platform for functional genomics studies and breeding applications.

## 1 Introduction

Wheat is one of the oldest and most widely cultivated crops. As a vital staple food, it supplies a large share of the daily energy, fiber, and essential micronutrients required for human nutrition. Globally, the dynamics of wheat production and trade significantly influence the international political and economic landscapes.

Mutation breeding induces heritable genetic variations via mutagenic agents to select improved crop varieties with traits such as disease resistance and high yield (Sikora et al., 2011; Pathirana, 2011). Radiation mutagenesis employs high-energy radiation to induce genetic alterations (Ma et al., 2021). This is of significant value for crop breeding and fundamental research (Geras’kin et al., 2025).

^60^Co-γ, an artificial radioactive isotope, decays by emitting high-energy gamma rays (typically 1.17 and 1.33 MeV) with strong penetrating power, which can induce DNA damage (Vaughan et al., 1991). As a type of ionizing radiation, these rays cause cellular damage in plants through direct ionization and indirect free-radical effects. Subsequent activation of DNA repair pathways may lead to imprecise repair and the introduction of point mutations and indels (Ali et al., 2015; Piri et al., 2011; Wang et al., 2022a; Kowalczykowski, 2015). Severe lesions (e.g., double-strand breaks) that remain unrepaired or misrepaired can cause extensive genetic alterations or cell death (Morgan et al., 1998; Obe and Durante, 2010; Pfeiffer et al., 2000). Heritable mutations transmitted via surviving reproductive or meristematic cells provide mutant lines for breeding selection (Barber et al., 2006), which typically include point mutations, indels, and structural variations (Alzu’bi et al., 2019).

As a response to radiation-induced damage, plant cells activate their defense and repair mechanisms to preserve genomic integrity, cellular viability, and overall survival (Kim et al., 2019). Immediate strategies involve the direct repair of DNA lesions, mitigation of oxidative stress, and coordinated cell cycle arrest to facilitate recovery. In the case of irreparable damage, programmed cell death is triggered to eliminate severely compromised cells (Greenberg, 1996; Pennell and Lamb, 1997). Plants sustain their developmental and reproductive functions through mechanisms such as protein homeostasis, epigenetic regulation, and tissue-level protection (Mahawer et al., 2022).

A key protective mechanism involves the activation of enzymatic systems to mitigate radiation-induced oxidative stress. Radiation primarily triggers water radiolysis, generating large amounts of reactive oxygen species (ROS) that cause oxidative damage (Lehnert and Iyer, 2002). For example, wheat seedlings exposed to ^60^Co-γ irradiation exhibit significantly enhanced superoxide dismutase (SOD), catalase (CAT), and ascorbate peroxidase (APX) activities, forming a coordinated defense system. Specifically, SOD dismutates the superoxide anion (O_2_^−^) into hydrogen peroxide (H_2_O_2_), which is then degraded by CAT and APX to prevent cytotoxic accumulation (Azarabadi et al., 2017; Ighodaro and Akinloye, 2018). Importantly, the induction of this enzymatic defense depends on the radiation dose (Prasannath, 2017).

In this study, ten wheat cultivars from Shandong, Henan, and Hebei were selected for ^60^Co-γ mutagenesis, and a 10,350,000 M_2_ mutant population, which serves as a valuable germplasm resource, was constructed. This population was specifically designed to support trait identification and the development of biotechnological wheat breeding.

## 2 Materials and methods

### 2.1 Plant materials

In this study, we selected ten wheat varieties from Shandong (‘Shannong 28,’ ‘Luyan 128,’ ‘Jimai 44,’ ‘Jimai 38,’ and ‘Yannong 1212’), Henan (‘Zhongmai 578,’ ‘Malan 1,’ and ‘Bainong 4199’), and Hebei (‘Zhongxinmai 998’ and ‘Gaoyou 5766’). In production, ‘Shannong 28,’ ‘Luyan 128,’ ‘Jimai 38,’ ‘Yannong 1212,’ ‘Malan 1,’ ‘Bainong 4199,’ and ‘Zhongxinmai 998’ are high-yielding varieties, and ‘Jimai 44,’ ‘Zhongmai 578,’ and ‘Gaoyou 5766’ are strong-gluten quality varieties.

### 2.2 ^60^Co-γ mutagenesis and mutant planting

A ^60^Co-γ radiation source provided by the Shandong Irradiation Center (Jinan, Shandong Province, China) was used for the experiment. Thirteen radiation dose levels were set: 0, 100, 200, 300, 400, 500, 600, 700, 800, 900, 1000, 1100, and 1200 Gy. For each wheat cultivar, 500 g of seeds was irradiated at each dose level. After irradiation, 200 seeds per cultivar (per dose) were sown in a greenhouse. The growth conditions were controlled at a constant temperature of 25 °C, 16/8 h light/dark cycle, light intensity of 300 μmol m^–2^ s^–1^, and relative humidity of 70%. After 14 days, germination and seedling growth were evaluated. Each treatment was conducted in triplicate. The formulas used to calculate the germination and normal growth rates are as follows:


Germination rate (%)=(Number of seedlings ≥2 cm in height/Total number of sown seeds) × 100

Normal shoots rate (%)=(Number of main shoots ≥20 cm in height/Number of seedlings ≥2 cm in height) × 100

Normal growth rate (%)=(Germination rate (%)×Normal shoot rate (%)) × 100

Efficient mutagenesis is typically associated with a normal growth rate of 20-40%. Based on this criterion, 100 kg of seeds per cultivar was subjected to ^60^Co-γ radiation mutagenesis for subsequent experiments. To balance the mutagenesis efficiency and population scale requirements, a gradient irradiation scheme centered on the optimal mutagenic dose, with ±50 Gy variations around this dose, was designed. Field sowing was performed at 225 kg ha^-1^, covering 0.67 ha per cultivar in October 2021 (**Supplementary Figure S1**). Given the large size of the mutant population, we pooled the M_1:2_ seeds separately for each cultivar in June 2022. The M_2_ mutant population served as the material for subsequent phenotypic characterization.

### 2.3 Identification of mutant phenotypes

In November 2022, Changqing experienced unseasonably warm conditions with an average temperature of 10 °C, followed by a cold wave from November 30 to December 3, during which temperatures decreased rapidly by 13-15 °C to a low of -5 °C. Severe frost damage occurs owing to the lack of cold acclimation in wheat under warm conditions. This natural frost event created a field-based selection environment for the identification of frost-tolerant mutants.

In October 2022, a mutant population (2 ha) was established in Kenli, Shandong Province. The experimental site featured saline-alkaline soil. The basic physicochemical properties of the soil are as follows: pH value ranges from 7.2 to 8.0; the soil ion composition includes Na^+^ content of 3.2-5.6 g kg^-1^, CO_3_^2-^ content of 0 g kg^-1^, and HCO_3_^−^ content of 1.6-1.9 g kg^-1^. Phenotypic screening for salt tolerance was conducted during the seedling (March) and maturity (June) stages in 2023.

### 2.4 Determination of ROS and antioxidant enzyme

The sampling site for ROS and antioxidant enzyme determination is specified as the first true leaf of wheat seedlings, and the sampling time is 14 days after radiation treatment (consistent with the seedling growth evaluation time).

For O_2_^−^ measurements, the samples were homogenized in a pre-chilled 50 mM potassium phosphate buffer (pH 7.8). The homogenate was mixed with 50 mM phosphate buffer (pH 7.8) and 10 mM hydroxylammonium chloride at a volume ratio of 1:1:2, incubated at 25 °C for 20 min, and subsequently mixed with twice its volume of ethyl ether. After absorbance quantification at 530 nm (Hao et al., 2018), the H_2_O_2_ content was determined following the method described by Mukherjee and Choudhuri (1983).

The activities of the antioxidant enzymes SOD (Wang et al., 2020) and CAT (Wang et al., 2022b) were determined following previously reported methods. SOD activity assay: The reaction system contains 50 mM phosphate buffer (pH 7.8), 13 mM methionine, 75 μM nitroblue tetrazolium (NBT), 10 μM EDTA-Na_2_, and 2 μM riboflavin. The reaction is carried out at 25 °C under a light intensity of 4000 lx for 20 min, and the absorbance is measured at 560 nm. One enzyme activity unit (U) is defined as the amount of enzyme required to inhibit NBT photoreduction by 50%; CAT activity assay: The reaction system contains 50 mM phosphate buffer (pH 7.0) and 20 mM H_2_O_2_. The reaction is performed at 25 °C, and the change in absorbance (ΔA_240_) is measured at 240 nm. One enzyme activity unit (U) is defined as the amount of enzyme that decomposes 1 μmol of H_2_O_2_ per minute. All samples were analyzed using a UV-2550 spectrophotometer (Shimadzu, Kyoto, Japan).

## 3 Results

### 3.1 Determination of the optimal ^60^Co-γ radiation dose for mutagenesis in ten wheat varieties from Shandong, Henan, and Hebei

**Figure 1:**
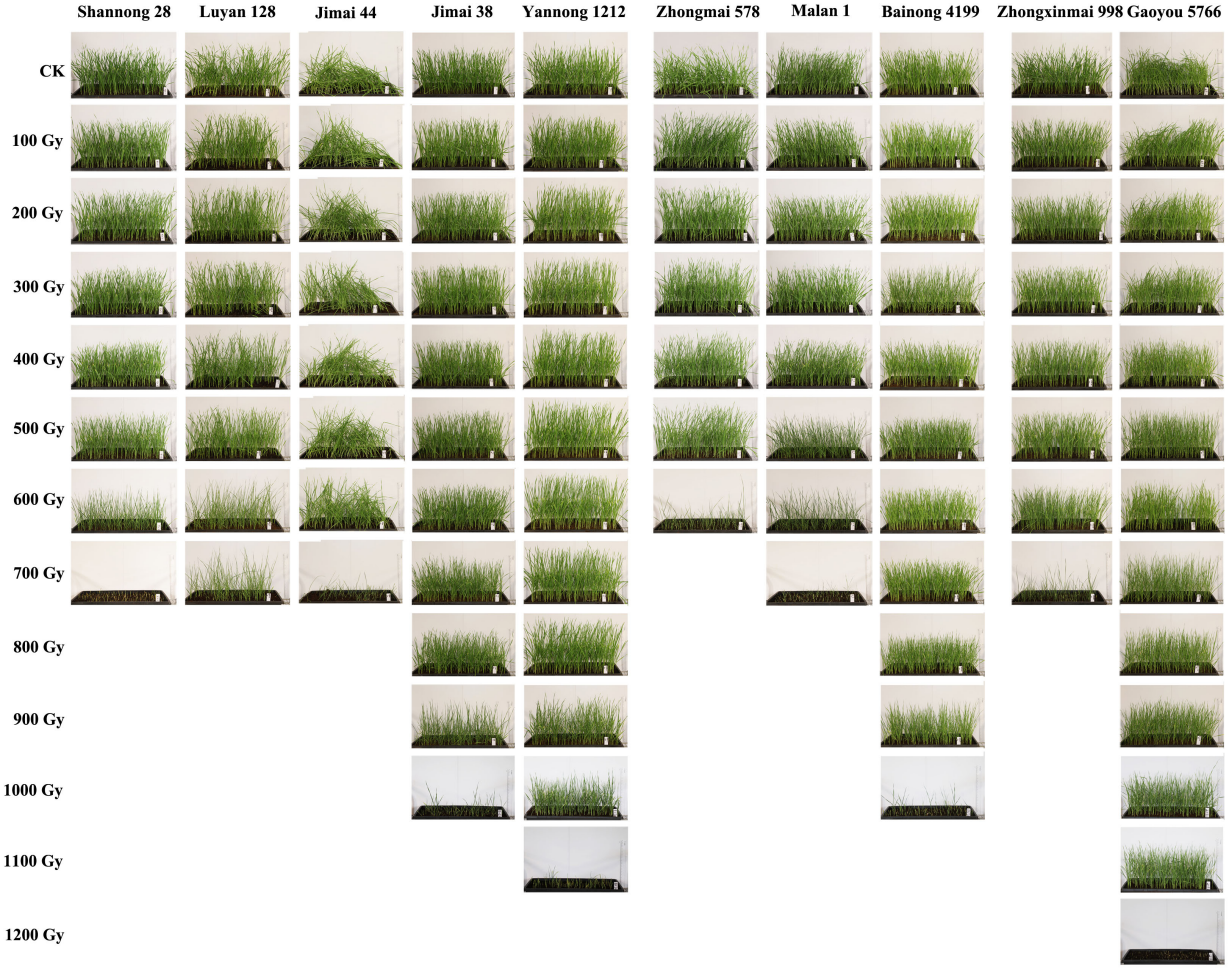
The effect of different ^60^Co-γ radiation dose on the growth in ten wheat varieties from Shandong, Henan, and Hebei.

In a preliminary experiment, ten wheat varieties from Shandong, Henan, and Hebei were exposed to different doses of ^60^Co-γ radiation. Seedling growth was evaluated 14 days after the seeds had germinated. The results showed a dose-dependent reduction in the normal growth rate, with significant varietal differences in radiosensitivity among the genotypes (**Figure 1**). At irradiation doses ≤400 Gy, all varieties grew normally. When the dose reached ≥600 Gy, the normal growth rates of radiation-sensitive varieties, including Shannong 28, Luyan 128, Jimai 44, Zhongmai 578, Malan 1, and Zhongxinmai 998, significantly decreased, whereas those of highly tolerant varieties, including Jimai 38, Yannong 1212, Bainong 4199, and Gaoyou 5766, only showed a marked decline at doses ≥900 Gy. Subsequently, the irradiation intensity that yielded a 20–40% normal growth rate was selected for the follow-up experiments. The respective ^60^Co-γ radiation doses and corresponding normal growth rates of the varieties were as follows: Shannong 28 (600 Gy, 28.8%), Luyan 128 (700 Gy, 26.6%), Jimai 44 (700 Gy, 25.4%), Jimai 38 (900 Gy, 27.5%), Yannong 1212 (1000 Gy, 33.7%), Zhongmai 578 (600 Gy, 25.5%), Malan 1 (600 Gy, 29.5%), Bainong 4199 (900 Gy, 31.6%), Zhongxinmai 998 (700 Gy, 22.9%), and Gaoyou 5766 (1100 Gy, 35.5%) (**Table 1**).

**Table 1:** The effect of different ^60^Co-γ radiation dose on the germination and normal growth rate in ten wheat varieties from Shandong, Henan, and Hebei^a^.

Cultivar	Plant responses	CK	100 (Gy)	200 (Gy)	300 (Gy)	400 (Gy)	500 (Gy)	600 (Gy)	700 (Gy)	800 (Gy)	900 (Gy)	1000 (Gy)	1100 (Gy)	1200 (Gy)
Shannong 28	Germination (%)	95.0	93.0	91.0	90.5	88.5	86.5	75.0	63.0					
	Normal growth (%)^b^	93.9	91.7	89.8	84.6	72.6	61.4	28.8	0					
Luyan 128	Germination (%)	95.5	95.0	94.5	93.0	92.0	90.5	86.0	76.5					
	Normal growth (%)	94.2	92.7	88.7	80.7	75.3	62.7	48.8	26.6					
Jimai 44	Germination (%)	93.5	93.0	90.5	89.5	88.0	87.5	86.0	65.0					
	Normal growth (%)	91.8	90.2	89.4	88.3	82.2	76.1	63.2	25.4					
Jimai 38	Germination (%)	95.5	94.0	93.5	92.0	90.5	90.0	88.5	86.5	85.5	83.5	76.0		
	Normal growth (%)	86.4	85.1	82.2	76.7	71.5	65.2	61.0	54.5	45.7	27.5	16.4		
Yannong 1212	Germination (%)	97.5	95.5	95.0	93.5	93.0	92.0	91.0	89.5	87.0	86.0	78.5	71.0	
	Normal growth (%)	95.3	94.6	92.9	89.7	86.7	84.5	80.1	76.4	71.6	49.4	33.7	7.0	
Zhongmai 578	Germination (%)	95.5	95.0	94.0	92.5	91.0	88.5	74.5						
	Normal growth (%)	94.3	92.9	90.1	86.7	78.6	66.8	25.5						
Malan 1	Germination (%)	97.0	94.5	92.5	90.0	88.5	86.5	75.5	67.5					
	Normal growth (%)	95.1	92.7	89.2	86.7	78.6	62.4	29.5	3.2					
Bainong 4199	Germination (%)	96.0	95.5	94.5	93.0	93.0	91.5	90.0	88.5	86.5	75.5	66.5		
	Normal growth (%)	94.5	93.5	93.0	91.8	90.4	89.5	82.1	78.2	66.7	31.6	7.8		
Zhongxinmai 998	Germination (%)	95.5	95.0	94.0	92.5	91.0	90.5	85.5	73.5					
	Normal growth (%)	93.0	91.9	88.1	87.7	81.6	76.8	68.5	22.9					
Gaoyou 5766	Germination (%)	94.5	93.5	92.0	91.5	90.5	90.0	88.5	87.5	86.5	85.0	84.0	80.5	72.5
	Normal growth (%)	93.5	92.1	90.9	90.0	88.4	86.2	83.4	81.9	77.6	71.5	64.3	35.5	0.0

a Plants were evaluated 14 days post-germination of 200 seeds for each ^60^Co-γ treatment;

b Germination with growth ≥ 20 cm above the soil.

### 3.2 The effect of ^60^Co-γ radiation mutagenesis on antioxidative competence in ten wheat varieties from Shandong, Henan, and Hebei

**Figure 2:**
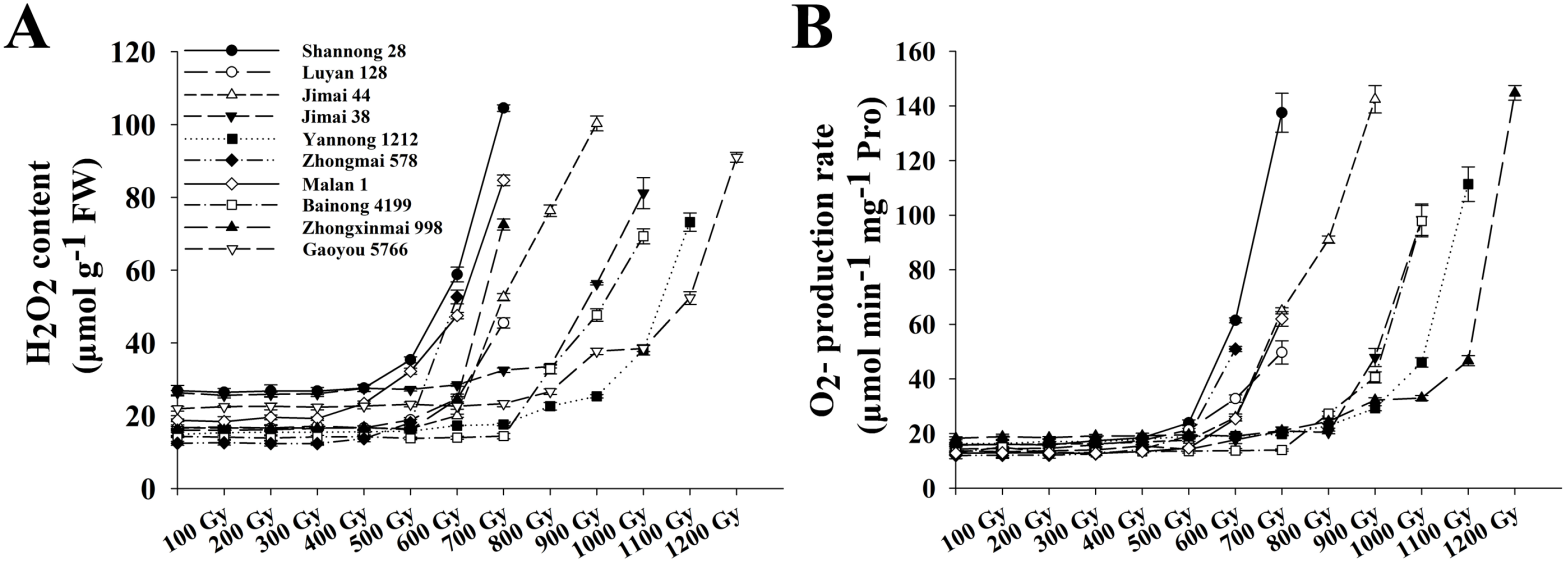
Changes in reactive oxygen species (ROS) accumulation under different ^60^Co-γ radiation dose in ten wheat varieties from Shandong, Henan, and Hebei. **(A)** H_2_O_2_ content; **(B)** O_2_^−^ production rate. Values are means ± SD of three replicates. Error bars indicate standard deviations.

**Figure 3:**
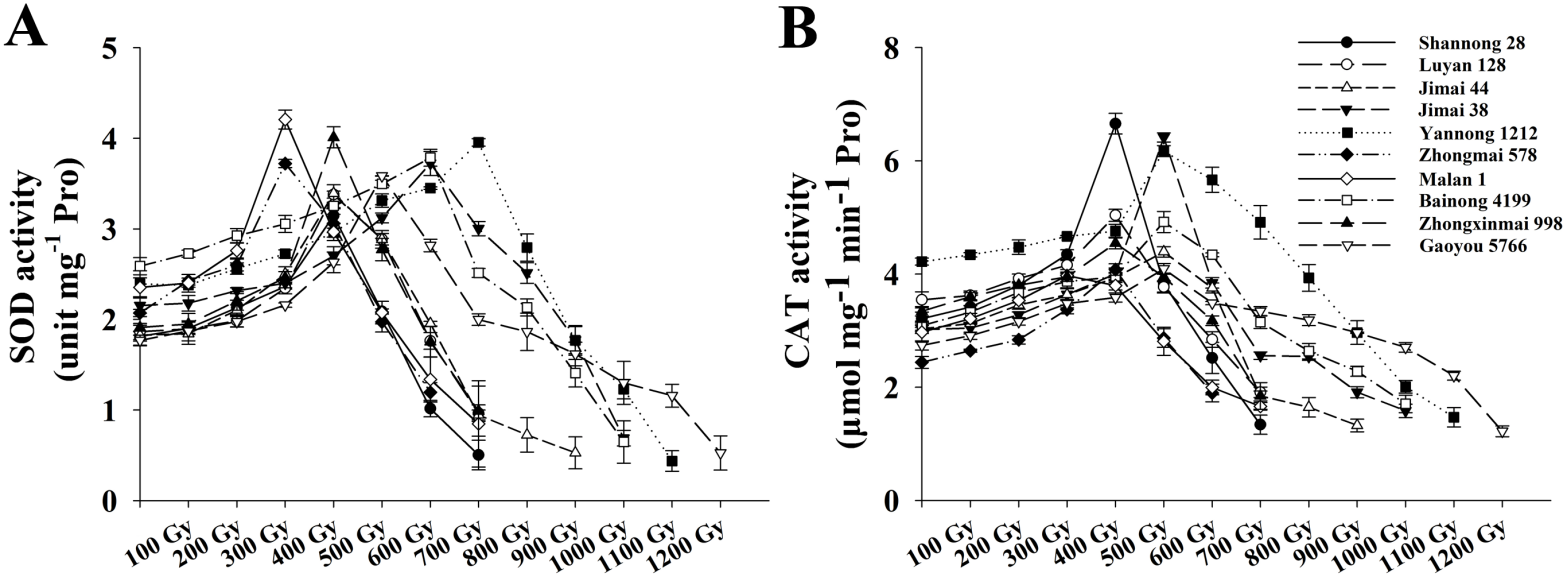
The effect of different ^60^Co-γ radiation dose on antioxidant enzyme activity in ten wheat varieties from Shandong, Henan, and Hebei. **(A)** SOD; **(B)** CAT. Values are means ± SD of three replicates. Error bars indicate standard deviations.

ROS accumulation and antioxidant enzyme responses were analyzed in the irradiated wheat seedlings. At doses ≤400 Gy, the H_2_O_2_ content (**Figure 2A**) and O_2_^−^ generation rate (**Figure 2B**) remained consistently low, with minor fluctuations, whereas SOD (**Figure 3A**) and CAT (**Figure 3B**) activities increased steadily with increasing doses. At ≥600 Gy, radiation-sensitive cultivars, including Shannong 28, Luyan 128, Jimai 44, Zhongmai 578, Malan 1, and Zhongxinmai 998, exhibited a sharp increase in H_2_O_2_ and O_2_^−^; the SOD and CAT activities peaked at 300–400 Gy, then declined progressively. In contrast, highly tolerant cultivars, such as Jimai 38, Yannong 1212, Bainong 4199, and Gaoyou 5766, exhibited a significant increase in H_2_O_2_ and O_2_^−^ at doses ≥900 Gy. Correspondingly, SOD and CAT activities were maximized at 500–700 Gy, followed by a steady decrease in activity.

### 3.3 Screening the freezing tolerance, saline-alkali tolerance, yield, and quality mutants in M_2_ populations

#### 3.3.1 Freeze tolerant mutants

**Figure 4:**
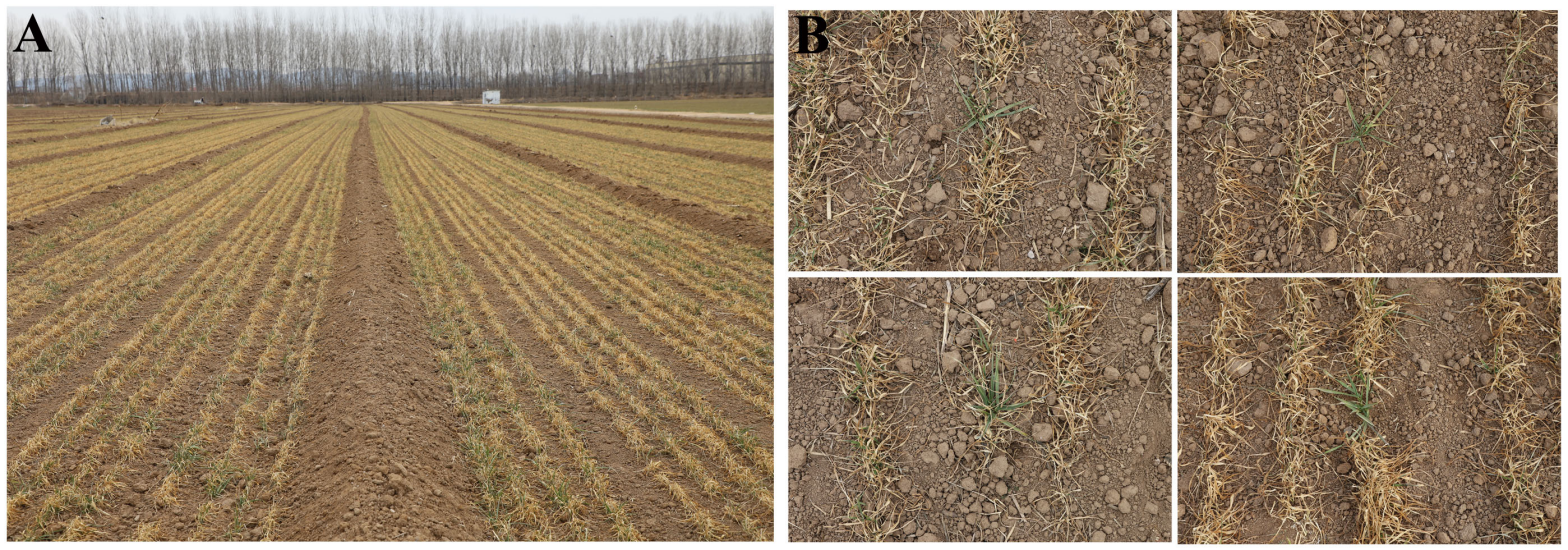
Identification of freeze tolerant mutants in the M_2_ populations. **(A)** Field phenotype; **(B)** Examples of mutants with freezing tolerance.

The Huanghuaihai Plain is one of China’s key wheat-maize rotation areas. In modern wheat breeding, semi-winter genotypes are widely used to balance high-yield potential and suitable phenology. In 2022, wheat crops lacked cold acclimation, and a rapid temperature drop occurred between November 30 and December 3. Frost damage creates a field-based environment for screening frost-tolerant mutants. Field observations showed that commercial cultivars, such as Jimai 38, Luyan 128, and Bainong 4199, suffered grade 4–5 freezing damage (Leaf damage area > 70%, extensive yellowing/whitening, survival rate of effective tillers < 30%, browning of injured tillers, softening and rotting of tissues, and eventual death) with extensive leaf chlorosis or whitening. In these susceptible lines, the injured tillers turned brown, with tissue softening and rotting, eventually leading to plant death. In contrast, freeze-tolerant mutants were identified by vigorous tillering and minimal symptom development, typically limited to yellowing or desiccation of the leaf tips or upper leaves, corresponding to grade 1 freezing tolerance (Leaf damage area < 10%, only slight yellowing/wilting at the leaf tips or upper leaves, survival rate of effective tillers ≥ 90%, and regrowth within 10 days after the cold wave) (**Figure 4**). We obtained 158 freezing-tolerant mutants during the 2023 growing season.

#### 3.3.2 Saline-alkali tolerant mutants

Soil salinization is a worsening challenge that severely compromises sustainable global crop production (Van Zelm et al., 2020). Wheat is highly sensitive to saline–alkali stress and suffers from Na^+^ toxicity under high-salinity conditions, leading to significant yield losses (Guo et al., 2009, 2015). A mutant population was planted in saline–alkaline soil (Na^+^ content: 3.2-5.6‰) covering an area of 2 ha to identify salt-tolerant germplasms.

**Figure 5:**
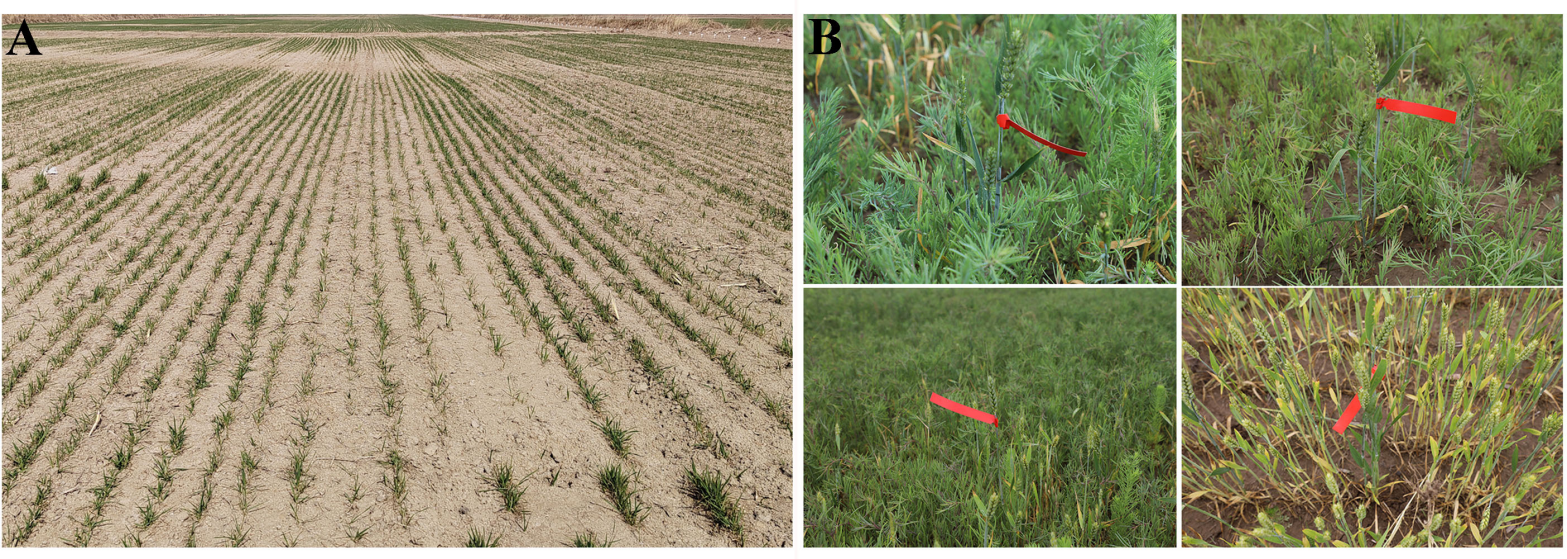
Identification of saline-alkali tolerant mutants in the M_2_ populations. **(A)** Field phenotype; **(B)** Examples of mutants with saline-alkali tolerance.

Under salt stress, most plants show typical injury symptoms at the seedling stage, including stunted growth, drastically reduced tillering (often only one tiller per plant), smaller leaf area, and necrotic scorching of leaf tips and margins (resembling leaf burns). At maturity, the stressed plants exhibited shorter spikes, fewer grains per spike, shriveled kernels, and a significant reduction in 1000-kernel weight. In contrast, salt-tolerant mutants were selected based on their ability to maintain multi-tillering capacity, retain leaf greenness, and develop larger and well-filled spikes under the same saline-alkaline conditions (**Figure 5**). During the 2023 growing season, we identified 441 saline–alkali-tolerant mutants.

#### 3.3.3 Yield and quality mutants

**Figure 6:**
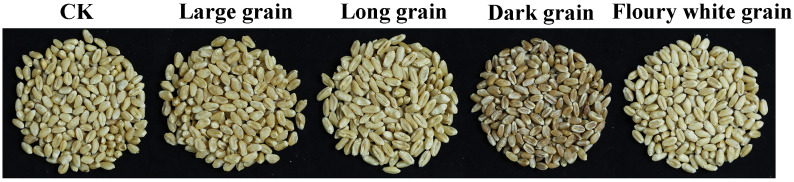
Examples of mutants with yield and quality in the M_2_ populations. CK is the original non-irradiated Jimai 44.

Grain size and quality are core phenotypic traits that determine wheat yield and market value. Grain size is typically evaluated using a 1000-kernel weight, which is affected by grain length, width, and plumpness (Gasparis and Miłoszewski, 2023; Wang and Sun, 2023; Xie et al., 2015). Quality traits include processing quality, which is largely determined by the storage protein content (Gao et al., 2021; Veraverbeke and Delcour, 2002; Varzakas, 2016), and nutritional quality, which encompasses the protein content, amino acid composition (particularly lysine), dietary fiber, vitamins, and mineral elements (Iqbal et al., 2022; Păucean et al., 2021). Grains from the M_2_ mutant population were assessed for yield and quality characteristics. Yield-related mutants were selected based on large- and long-kernel phenotypes, whereas quality-related mutants were identified by dark and floury white kernel coloration (**Figure 6**). More than 5,000 yield- and quality-related mutants were identified during the 2023 growing season.

### 3.4 Availability of wheat ^60^Co-γ M_2_ populations

In this study, a 10,350,000 ^60^Co-γ irradiated M_2_ mutant population was established, comprising the wheat varieties ‘Shannong 28’(1,010,000 lines), ‘Luyan 128’(1,420,000 lines), ‘Jimai 44’(1,730,000 lines), ‘Jimai 38’(1,080,000 lines), ‘Yannong 1212’(750,000 lines), ‘Zhongmai 578’(1,200,000 lines), ‘Malan 1’(940,000 lines), ‘Bainong 4199’(890,000 lines), ‘Zhongxinmai 998’(920,000 lines), and ‘Gaoyou 5766’(410,000 lines). The M_2_ generation exhibited the highest frequency of induced mutations, making it a critical genetic resource for wheat improvement. Currently, M_2_ bulk seed pools have been distributed to collaborative research programs to facilitate the screening of germplasm with desirable traits, including resistance to rust, powdery mildew, and drought tolerance.

## 4 Discussion

### 4.1 Achieving an effective ^60^Co-γ mutagenesis in wheat

The mutagenic efficiency of ^60^Co-γ radiation is determined by the synergistic interplay of multiple factors. Irradiation parameters, including the total dose, dose rate, and exposure mode, which collectively determine the extent of genetic damage and mutation frequency, should be precisely regulated (Lowe et al., 2022; Sikder et al., 2013). The inherent biological properties of irradiated materials are equally important because significant variations in radiosensitivity exist across species and cultivars. Concurrently, mutagenic efficiency is modulated by critical physiological parameters, such as seed moisture content, developmental stage, and oxygen availability (Duarte et al., 2023; Khare et al., 2025).

Early studies primarily focused on the median lethal dose (LD50), defined as the ^60^Co-γ radiation dose that kills 50% of treated seeds (Xiong et al., 2023; Yang et al., 2014). The LD50 criterion performs reasonably well in practice, as it consistently generates populations with a sufficient percentage of mutations. In the present study, we determined the optimal ^60^Co-γ dose, under which the normal growth rate of 20-40% under optimal germination conditions (**Figure 1**; **Table 1**).

In previous studies, irradiating dry wheat seeds with 200-400Gy of ^60^Co-γ radiation could achieve the half-lethal dose and obtain high mutation efficiency. This approach facilitates the selection of wheat germplasm with enhanced disease resistance, stress tolerance, high yield, and improved quality for breeding (Cheng et al., 2008; Wang et al., 2019; Xiong et al., 2023; Zhu et al., 2010). Although a rigorous dose-gradient experiment was conducted in the current study, the required mutagenic dose (with a normal growth rate of 20-40%) was significantly higher than that reported 200-400Gy previously. However, a repeated irradiation experiment was conducted on seeds of Jimai 38 in 2023, with a moisture content of 13.0% and a post-harvest storage period of 3 months. The radiation dose corresponding to a normal growth rate of 28.3% was 1100 Gy, suggested that the higher optimal radiation dose was due to the decay of the ^60^Co-γ radiation source over time. Nevertheless, germplasm screening was effective.

### 4.2 Improved antioxidative competence mitigates ^60^Co-γ radiation effects

Plants exposed to ^60^Co-γ radiation undergo a rapid surge in intracellular ROS, a phenomenon known as “oxidative burst” (Ksas et al., 2024). This occurs via two primary mechanisms: first, the direct radiolysis of water molecules generates ROS, such as hydroxyl radicals, O_2_^−^, and H_2_O_2_ (Wojtaszek, 1997); second, the disruption of intracellular electron transport chains in mitochondria and chloroplasts enhances electron leakage and subsequent ROS formation (Asada, 1999; Robertson et al., 1995).

Excessive ROS accumulation causes significant cellular damage by inducing membrane lipid peroxidation, compromising membrane integrity (Langebartels et al., 2002), promoting oxidative protein denaturation (Sood, 2025), and causing DNA strand breaks (Roldán-Arjona and Ariza, 2009). Plants activate endogenous antioxidant enzyme systems to mitigate oxidative stress (Cannea and Padiglia, 2025; Zheng et al., 2025).

In the present study, the antioxidant enzyme activity exhibited a characteristic biphasic response to increasing radiation intensity, with an initial enhancement followed by a gradual decline. Under low-dose irradiation, elevated enzymatic activity was essential for preserving cellular redox homeostasis by effectively scavenging ROS. However, beyond a critical radiation threshold, the compromised antioxidant system proved insufficient to neutralize excessive ROS accumulation, ultimately triggering an oxidative burst in wheat seedlings (**Figures 2**, **3**). This redox imbalance caused growth arrest and seedling mortality. Notably, ROS accumulation and antioxidant enzyme activity exhibited a clear dose-dependent relationship with radiation intensity.

### 4.3 Application value of ^60^Co-γ M_2_ populations

The ^60^Co-γ irradiation mutant library systematically constructed in this study comprised elite cultivars from Shandong, Henan, and Hebei, with 10,350,000 lines. The library offers extensive genetic diversity and supports multiple breeding objectives. For breeding applications, we identified mutants with improved freezing tolerance, saline-alkali resistance, yield potential, and quality traits (**Figures 4**-**6**). These mutants are currently being used in crossing programs to introduce desirable traits into advanced breeding lines. The mutant library provides valuable resources for gene cloning and mechanistic studies of stress tolerance, yield, and quality. In genomic research, exome capture sequencing enables the compilation of a genotype-phenotype association database to support the targeted selection of favorable mutations. Integrating these mutant resources into molecular design breeding frameworks will facilitate systematic trait pyramiding and accelerate the development of elite cultivars with superior agronomic performance.

## 5 Conclusion

This study systematically optimized ^60^Co-γ radiation mutagenesis in wheat by establishing a precise dose-response framework for ten wheat cultivars from Shandong, Henan, and Hebei, based on their distinct physiological responses. We generated a 10,350,000 M_2_ mutant population and applied an integrated natural selection system, which identified 158 freezing-tolerant mutants, 441 saline-alkali-tolerant mutants, and >5,000 mutants with changed yield or quality traits. This well-characterized genetic resource provides breeding materials ready for immediate use and creates a functional genomics platform for gene discovery and molecular mechanism analysis, thereby establishing a robust foundation for wheat improvement.

## Data Availability

The original contributions presented in the study are included in the article/**Supplementary Material**. Further inquiries can be directed to the corresponding authors.

## Supplementary material

Supplementary Figure 1 The growth condition of M_1_ generation under ^60^Co-γ radiation in ten wheat varieties from Shandong, Henan, and Hebei in the field.
