# Supplementary figures and images for: Creating large 60Co-γ populations for functional genomics and breeding in wheat
Source: Front Plant Sci. 2026 Jan 26;16:1760299. doi: 10.3389/fpls.2025.1760299 (PMC12885088; doi:10.3389/fpls.2025.1760299)

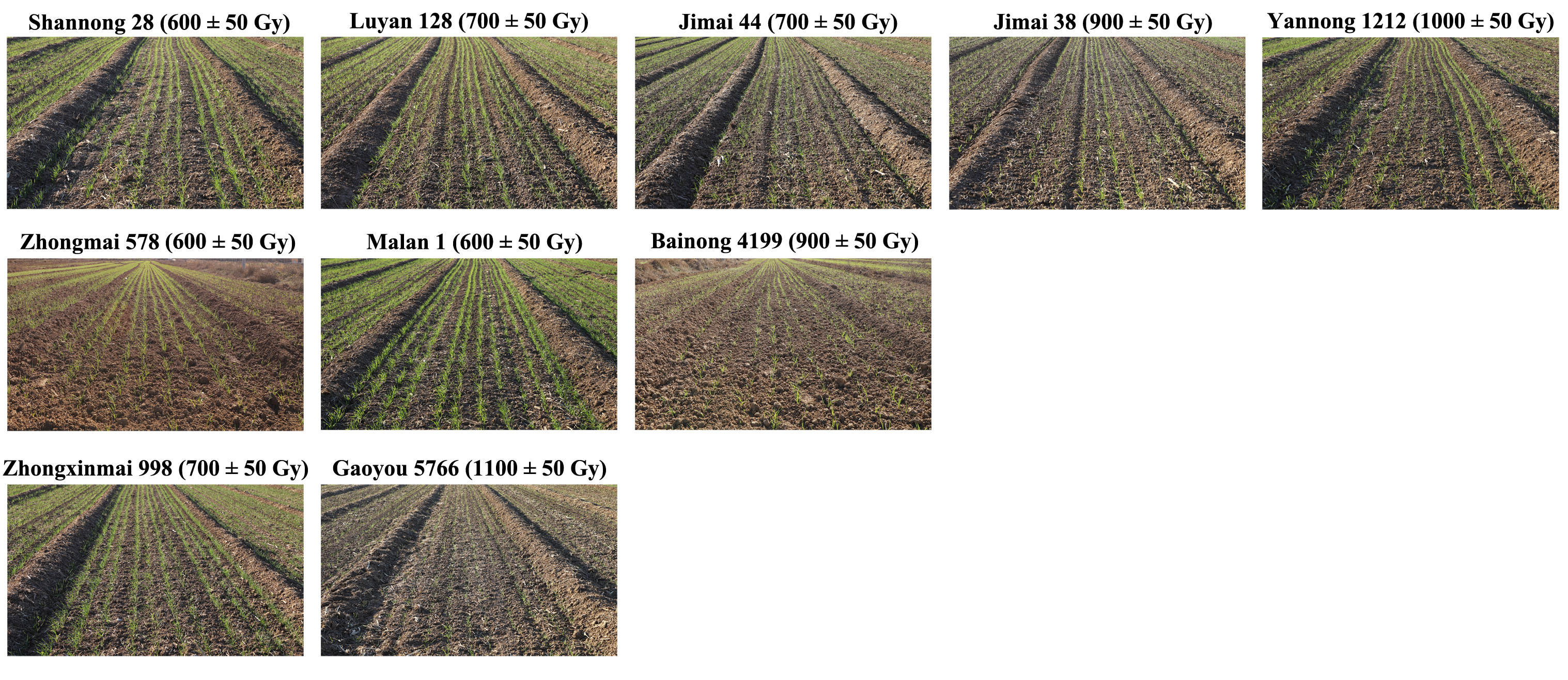

Supplement: Supplementary Figure 1 — The growth condition of M1 generation under 60Co-γ radiation in ten wheat varieties from Shandong, Henan, and Hebei in the field. [file Image1.jpeg]
